# The role of accountability in the performance of Jazia prime vendor system in Tanzania

**DOI:** 10.1186/s40545-020-00220-8

**Published:** 2020-06-08

**Authors:** August Kuwawenaruwa, Fabrizio Tediosi, Brigit Obrist, Emmy Metta, Fiona Chiluda, Karin Wiedenmayer, Kaspar Wyss

**Affiliations:** 1 Ifakara Health Institute, Plot 463, Kiko Avenue Mikocheni, P.O. Box 78 373, Dar es Salaam, Tanzania; 2 Swiss Tropical and Public Health Institute (Swiss TPH), Basel, Switzerland; 3 University of Basel, Basel, Switzerland; 4 The Muhimbili University of Health and Allied Sciences (MUHAS), School of Public Health and Social Sciences (SPHSS), Dar es Salaam, Tanzania; 5 Health Promotion and System Strengthening (HPSS) project, Dodoma, Tanzania

**Keywords:** Accountability, Medicines, Performance, Prime vendor system, Tanzania

## Abstract

**Background:**

Access to safe, effective, quality and affordable essential medicines for all is a central component of Universal Health Coverage (UHC). However, the availability of quality medicines in peripheral healthcare facilities is often limited. Several countries have developed integrated complementary pharmaceutical supply systems to address the shortage of medicines. Nevertheless, there is little evidence on how accountability contributes to the performance of such complementary pharmaceutical supply systems in low-income settings. The current study analyses how accountability mechanisms contributed to the performance of Jazia Prime Vendor System (Jazia PVS) in Tanzania.

**Methods:**

The study analysed financial, performance and procedure accountability as defined in Boven’s accountability framework. We conducted 30 in-depth interviews (IDIs), seven group discussions (GD) and 14 focus group discussions (FGDs) in 2018 in four districts that implemented Jazia PVS. We used a deductive and inductive approach to develop the themes and framework analysis to summarize the data.

**Results:**

The study findings revealed that a number of accountability mechanisms implemented in conjunction with Jazia PVS contributed to the performance of Jazia PVS. These include inventory and financial auditing conducted by district pharmacists and the internal auditors, close monitoring of standard operating procedures by the prime vendor regional coordinating office and peer cascade coaching. Furthermore, the auditing activities allowed identifying challenges of delayed payment to the vendor and possible approaches for mitigation while peer cascade coaching played a crucial role in enabling staff at the primary facilities to improve skills to oversee and manage the medicines supply chain.

**Conclusion:**

Financial, performance and procedure accountability measures played an important role for the successful performance of Jazia PVS in Tanzania. The study highlights the need for capacity building linked to financial and supply management at lower level health facilities, including health facility governing committees, which are responsible for priority-setting and decision-making at facility level.

## Background

Access to safe, effective, quality and affordable essential medicines for all is a central component of Universal Health Coverage (UHC) [1]. Yet the availability of quality medicines in low and middle-income countries (LMICs) is often limited especially in peripheral healthcare facilities [2–6]. Central Medical Stores (CMSs) are the main supplier of healthcare commodities to public healthcare facilities. However, CMSs are faced with several challenges, including inadequate resources, insufficient transparency, weak accountability mechanisms, inaccurate forecasting of medicines at the facility and national level, thefts, and ineffective systems for fulfilling back-ordered items [7–9]. Wirtz et al. highlighted other challenges such as the existence of substandard and falsified medicines, affordability, and inefficiencies in medical prescriptions to the patients (leading to overuse, underuse, and incorrect use) [1, 10].

Several countries have implemented initiatives to strengthen the medical supply systems through collaborations between the public, non-governmental, and commercial sectors [11–13]. For instance, in some countries governments contracted private companies as a prime vendor under public-private partnership (PPP). Health facilities (or districts) purchase medical commodities from the prime vendor, usually a single local wholesaler, at agreed prices, rather than purchasing directly from the various manufacturers [14, 15]. Weaver et al (1994) found an increase in drug availability after implementation of the prime vendor system within the Department of Veterans Affairs (VA) hospitals in the United States of America (USA) [16]. Furthermore, the prime vendor system was associated with faster turnaround, higher order fulfilment rates, costs reduction and increased satisfaction among program users [17]. For example, in Zambia, such contractual agreements between the government and the vendors lead to higher flexibility in quantities ordered as well as delivery schedules, together with improved availability of medicines, and decreased stock-outs [18].

In 2001, the Evangelical Lutheran Church (ELCT) of Tanzania implemented a prime vendor system, under the Mission for Essential Medical Supplies (MEMS), to complement the existing medical supply chain [19, 20]. The MEMS failed to meet the contractual terms as it underestimated the program complexity, leading to low coverage and over-reliance of the program on donor funding [20]. Two years later, another PPP was launched in Tanzania to improve access to medicines in peripheral areas through the accreditation of drug dispensing outlets (ADDOs) program [11]. In 2011 the Tanzanian government, with funding from the United States President’s Emergency Plan for acquired immune deficiency syndrome Relief (PEPFAR) [21], launched a prime vendor model. This model relied on the existing local pre-selected pharmaceutical vendors for the purpose of addressing gaps for 45 opportunistic infection medicines [21].

**Fig. 1:**
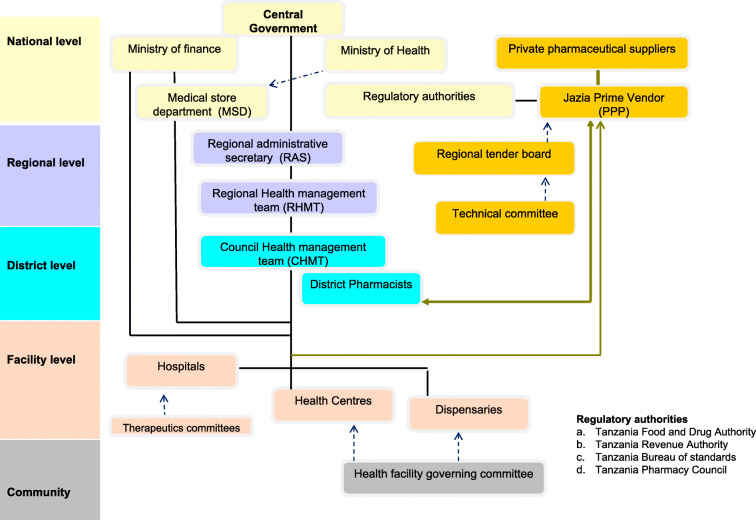
Prime vendor operation structures

In 2014, the regional authorities in Dodoma, Morogoro and Shinyanga regions started implementing a complementary pharmaceutical supply system, the Jazia Prime Vendor system (Jazia PVS) (see supplementary material Box S1, Fig. 1), with the support of the Health Promotion and Systems Strengthening (HPSS) Project [22]. The Jazia PVS is a PPP that complements national Medical Stores Department (MSD) with supplies from a single private contracted vendor, in a pooled regional approach. Jazia PVS is anchored in the structures of the regional health administration and it is overseen, supported and managed by mandated administrative structures such as regional administrative secretary, regional prime vendor coordinating office, regional health management teams and council health management teams. On-going monitoring and evaluation reports show that Jazia PVS has been successful in improving the availability of medicines, equipment and supplies at the peripheral healthcare facilities within the pilot regions [22–25]. Jazia PVS has reportedly improved the availability of essential medicines in primary health facilities from an average of 69% in 2014 to 94% in 2018 [23]. Improved availability of medicines at peripheral healthcare facilities is closely associated with a better quality of healthcare services (as perceived by patients), which in turn creates trust in public institutions [26]. As a consequence, the government of Tanzania decided to roll out Jazia PVS in other regions since October 2018 [23, 27]. However, key accountability aspects that contributed to Jazia PVS performance have not been assessed yet.

Accountability mechanisms are important for maintaining high availability of essential medicines while at the same time containing drug costs [2, 9, 28]. Effective accountability in the medicines supply chain has been shown to reduce inefficiencies in supply management, corruption, unethical practices [fraud and abuse], and rate of falsified and substandard medicines in the system [2, 4, 29]. This manuscript analysesaccountability mechanisms contributing to the performance of Jazia PVS in Tanzania. Understanding such accountability mechanisms provides evidence to inform policy-decisions on how to boost the performance of the system in general.

## Methods

### Theoretical framework

Accountability has been conceptualized in different ways [30–32] and it is often related to several concepts such as responsiveness, responsibility, and effectiveness [31]. Accountability refers to the obligation to explain and justify conduct [30–32]. In this study, we adapted the concept of accountability used by Boven to analyse the accountability of transnational institutions such as the European Union [31]. According to this conceptualization, accountability is described as *“a relationship between an actor and a forum, in which the actor has an obligation to explain and to justify his or her conduct, the forum can pose questions and pass judgment, and the actor may face consequences”* [31]. Such relationships between the actor and a forum give rise to a number of accountability classifications which are linked in some way [31–33]. Following Boven’s framework, we explored three of these accountability dimensions: financial, performance, and procedure accountability (Table 1). Financial accountability entails an actor’s compliance with various rules, laws and regulations concerning financial control and management. It is related to the responsibility for tracking and reporting on resource allocations, disbursements, and decisions on the utilization of financial resources in accordance with laws, rules, and regulations on financial control and management. Performance accountability refers to the accomplishment of an agreed-upon set of performance indicators and targets. We focused on district managers’, vendors’, and providers’ efforts in achieving performance targets set within the Jazia PVS. Procedure accountability finally refers to the processes used to arrive at pre-determined performance targets [34]. The focus here was on the strategies taken by facility and district managers to arrive at desired program targets.

**Table 1:** Themes, codes and sub-codes

Main themes	Codes categorization	Inductive-codes
Financial accountability	Revenue tracking and reporting	• Direct health facility financing • Facility bank accounts • Cost-sharing funds at facility
	Tracking and reporting on funds utilization	• Review of facility documents • Decision-making process • HFGC meetings • Itemization of facility needs
	Financial and inventory auditing	• Standardized auditing tools • Document review at facility • Healthcare commodity labelling • Feedback meetings/reports
Performance accountability	Medicines availability	• Quarterly medicines availability assessment • Facility staff experience
	Delivery time of consignment	• Number of days taken to deliver consignment • Transportation and communication
Procedure accountability	Adherence to standard operating procedures	• Facility quantification • Delivery of the consignment • Inspection of the consignment • Payment to the vendor • Communications channels
	Payment terms of the vendor	• Contractual agreement on payments to the vendor • Communications channels • Document reviews
	Peer cascade coaching	• Coaches selection process • Training on integrated logistic supply • Facility allocation for coaching • Content and frequency of coaching
	Redistribution of medicines	• Role of supportive supervision • Documentation in the facility

### Study settings

The study was conducted by a team of independent researchers in four districts that implemented Jazia PVS: Kondoa and Bahi district (of Dodoma region) and Ulanga and Kilosa districts (of Morogoro region). Districts selection was based on documented prime vendor volume use and distance from the regional Jazia PVS coordinating office. Two districts with high volume use of the Jazia PVS and two other districts with low volume use of the prime vendor were included in the study. Jazia PVS monitoring and evaluation reports showed that over 18 months of implementation Kilosa and Kondoa districts had high volume use of the prime vendor while Bahi and Ulanga had low volume use of Jazia PVS. The study districts counted a total population of 1,194,727, with Ulanga having the largest population (438,175, 36.7%) while Bahi had the smallest population (221,645, 18.6%). The study included a total of 27 purposively-selected health facilities (Table 2), roughly 20% of all public health facilities in these districts. The facilities were selected in collaboration with the regional and district managers based on documented Jazia PVS use by facilities and proximity to the district headquarters.

**Table 2:** Key characteristics of the study settings

District council variable	Kondoa	Bahi	Ulanga	Kilosa
Population^1^	269,704	221,645	265,203	438,175
Area coverage in square kilometres	5921	5948	24,460	14,918
Number of hospitals	1	0	1	1
Number of public health centres	2	6	2	6
Number of public dispensaries	27	35	43	19
Number of private facilities	12	2	6	25
Number of primary care facilities per 10,000 population	1.6	1.9	1.9	1.2
Facility sampled	7	6	7	7
Wards	28	20	24	35
Villages	107	57	68	143

^1^Presents information from the study districts. Information on the population was obtained from the Tanzania National Bureau of Statistics; Population and Housing Census 2013. While information on facilities was obtained from the Tanzanian national health facility registry portal

### Study design and population

This was a qualitative exploratory study. Data were collected via 30 in-depth interviews (IDIs), 7 group discussions (GDs) and 14 focus group discussions (FGDs) between July and September 2018. The FGDs lasted between 1 h and a half to 2 h, while GD and IDI lasted between 45 min to 1 h. FGDs participants had a median age of 40.5 years, and the majority (59/80) had attended lower level schools (primary and secondary). While GD participants had a median age of 43.5 years, and the majority (13/18) had attained education above the secondary level.

IDIs were used to explore accountability mechanisms in-depth with purposively selected study participants, namely Council Health Management Team (CHMT) members, district internal auditors, procurement managers; healthcare providers; council health service board (CHSB) members; regional health management team (RHMT) members; representatives from the President’s Office for Regional Administration and Local Government (PO-RALG); personnel of the Jazia PVS regional coordination office; a representative of the Prime Vendor; Jazia PVS Consultant and HPSS regional managers.

To capture diverse experiences and discuss controversial issues at the healthcare facility and community interface we conducted FGDs with six – eight participants with the healthcare facility governing committee (HFGC). During fieldwork, we repeatedly faced the situation where the suggested number of participants could not be enrolled in a focus group discussion. Accordingly, in those instances where only less than 5 persons participated, renamed the discussion a group discussion given the discrepancy to the outlined size and interactions within a focus group. GD participants included purposefully selected healthcare workers, members of CHMT and the district HPSS project officers/assistants. See Tables S1 and S2 in supplementary materials for further details on study participants.

### Data collection

To achieve consistency and accuracy in translation of tools and data, two research assistants with a social science background and extensive training on qualitative methods at a postgraduate level assisted during translation of data collection tools, pilot, data collection and transcription of audio. The interview guides were developed in English and translated into the local language, Swahili. All discussions and interviews were conducted in Swahili and were audio recorded with the permission of the participants. Thereafter the first author reviewed all the translated documents. Translated documents were also reviewed by a senior social scientist to ensure the text is error-free and the content is the same based on the original document (s). All tools were piloted in two different study sites and modified where appropriate before the commencement of the actual data collection.

### Data management and analysis

Data were transcribed verbatim 48 h after being generated. This allowed for easy follow-up and clarification of issues as they emerged during data collection till we achieved data saturation. All electronic transcribed documents alongside the audio records were cross-checked for their quality assurance. The transcribed data from voice recordings were read and re-read to gain an initial impression of the data and an in-depth understanding of participants’ descriptions before developing the codebook and data analysis. All analyses were conducted on the Swahili language transcripts and only the verbatim key quotations incorporated in this manuscript were translated into English. The analysis was facilitated by the qualitative data management software NVivo 12.0 (QSR International Pty Ltd).

The analysis was conducted by first author and done on two levels. The first level entailed the development of inductive (ideas originating from the data itself) and deductive codes (theoretical understanding, empirical literature review together with several researchers’ experiences) (Table 1). Both inductive and deductive codes were developed by the first author in collaboration with the co-author EM; any discrepancies were reconciled among the two. The 1st author coded the data, analysed and drafted the manuscript. However, throughout the process received feedback and support from the senior scientists who are also co-authors on this manuscript. The second level of analysis involved classification and categorizing the codes at abstract level according to key concepts and emergent patterns. Different themes representing the accountability constructs of Boven’s framework have included both inductive and deductive categories. Framework analysis was adapted for summarizing the data to address the research questions [35, 36]. Validation of the study findings was done by triangulating and synthesizing data across respondent groups. Triangulation of data collection methods allowed for the observation of similarities and differences among and across different levels of the informants and in the districts. The approach allowed for an in-depth, multi-faceted exploration of complex issues in the participants’ real-life settings [37]. The results have been organized according to the Boven’s accountability framework (see Table 1). The themes include (i) financial, (ii) performance and (iii) procedure accountability.

## Results

### Financial accountability

#### Revenue tracking and reporting

We found that the Jazia PVS implementation was supported by health financing policy reforms that were under way in the study districts. First, these districts implemented the so-called direct health facility financing (DHFF^1^), whereby each healthcare facility has its own bank account. The implementation of DHFF was an important prerequisite for Jazia PVS to be successfully implemented because facilities have financial autonomy and flexibility in the use of funds held in their bank accounts. Second, the Community Health Fund (CHF) premium and the related government matching funds, along with the financial flows coming from the ‘health basket fund’ fed by various donors, and the National Health Insurance Fund (NHIF) facilitated Jazia PVS implementation. The health worker in-charge (the in-charge) of the facility mentioned the importance of receiving timely and regularly funds. They said that the health facility accounts are directly credited with funds from the basket fund on a quarterly basis, from the NHIF reimbursements on monthly basis, and from the CHF reimbursements on monthly basis. On monitoring the basket fund allocations one of the health facility in-charges noted the following:

“ … ..*each year as we prepare our facility budget there is an amount of basket fund that is allocated for us, so each quarter it is credited into our account and we are informed, we ask for the bank statement for verification and then we use the funds* … ..” IDI, Facility in-charge, Dodoma.

The health facility in-charges and their assistants are responsible for depositing the user fees into the health facility bank accounts on a monthly basis. The facility in-charges said that for the purpose of financial accountability they were supposed to present a banking slip to the HFGC when the funds had been deposited into the bank accounts. This is an important accountability mechanism whereby after receiving the bank slip the HFGC members conduct validation by checking whether the amount deposited into the account equals the amount collected in the receipts book. HFGC validates the amount on the deposit slip by reviewing information from monthly bank statements as well as from the financial books available at the facility. Facility in-charges reported maintaining records of all the clients visiting the facility, categorising them by different payments options (such as insured, waived and those paying out-of-pocket). During the HFGC meetings, they reviewed all the records in the book, deposit slip and bank statements. During discussion some of the HFGC members felt that they do not have much knowledge on issues related to health commodities and financial reports. This issue was also attested during the FGDs with the HFGC when they mentioned that they can see whether the amount deposited in the health facility accounts equals the account collected:

“ … ..*the person who deposited the money at the bank, must come with a bank slip which shows on a certain date the money was deposited and we observe whether the amount deposited is equal to the amount collected* … ..” FGD, HFGC, Dodoma.

#### Tracking and reporting on funds utilization

The decisions on how to allocate financial resources to purchase medical commodities are left to the HFGC and should be approved by the district manager. This committee sits and discusses the health facility needs, and it then makes the necessary decisions. As a way of accounting for the financial resources utilized at the facility, the health facility in-charge share the minutes of the HFGC meetings with the district manager for their approval. The HFGC involves in their decisions district pharmacists and the district medical officer (DMO), as explained by the district manager when elaborating on the process of financial accountability

“ … .*.they (HFGC members) sit in their meetings and identify what they need to purchase, then they bring the needs to the pharmacist who reviews facility needs and then shares with the DMO. The DMO review the money which the facility wants to use and compares the amount with revenue available in the facility bank account. Our role is to review and approve ..*..” IDI, District Manager, Morogoro

The importance of the role of the HFGC was confirmed by health workers at the healthcare facility, reporting that health workers cannot authorise the use of funds without HFGC approval. However, it is important to highlight that HFGC members do not have strong financial management skills such as budgeting, clear record accounting, monitoring, and reporting. Before the HFGC meeting, the health facility in-charge in collaboration with other staff identifies and shortlists all the medical commodities to be purchased from the prime vendor and their respective monetary value. This process emerged clearly during the HFGC discussions:

“ … .. … *together with colleagues (other staff) at the facility, we review the availability of medicines, we compare medicines received against the quantity we ordered from (MSD). For example, if in the order we did not receive ten tins of Amoxicillin, we document for each item missed from MSD, together with their respective (vendor) prices. I then inform the HFGC. Once they authorise, I submit the meeting minutes, the request and the reporting form for the vendor to the district pharmacist …* ..” FGD, HFGC, Morogoro

#### Financial and inventory audits

Financial and inventory audits are key to strengthening management and implementation capacity of healthcare intervention and are supposed to be done quarterly for each health facility. Financial and inventory audits are done both as regular and unannounced activity as part of supportive supervision. District internal auditor leads financial audit, accompanied by other CHMT members, using standardized auditing tools. They review all the documents used for authorising procurement and payments to the vendor, whether they were procured from the contracted prime vendor. They also examine whether approval documents were submitted to the district managers and whether the prices paid for the purchases were in line with the contractual agreement. When discussing audits, the district managers said:

“ … .*. As an internal auditor, we conduct financial audits for the user fees, we audit the way in which medicines equipment and medical supplies were purchased from the vendor....*..” IDI, District Manager, Morogoro

After auditing, health facility staffs are given feedback by auditors and reports are shared with other district managers for close follow-up. In the case of inconsistencies and unethical practices, facility in-charges are instructed to reimburse funds which have been identified to be misused and the in-charges are given warnings.

District pharmacists lead the inventory audits with the support from other CHMT members. Pharmacists reported that a minimum of 24 essential medicines and related transactions are audited using a uniform tool which was prepared for the monitoring of the prime vendor system across the region. Together they review ledgers, bin cards, dispensing registers, issued vouchers and purchase requisition documents for the previous 3 months before the visit date. Also, they reported that review teams interviewed staff responsible for the medicine supply chain at each health facility. According to the health workers, medical commodities procured through the prime vendor are audited more regularly than those supplied via MSD. This is because medical commodities procured via the vendor were not labelled compared to those from MSD labelled with “*GOT (Government of Tanzania) and MSD logo*”. Labelling is intended to differentiate MSD supplies from other medicines found in the private hospitals and pharmacies [38], to mitigate the risk of unethical practices such as fraud, abuse and sale to the local drug dispenser outlets. Document review also ensures that facilities are using the prime vendor system. CHMT members use a drug-tracking tool to review ledgers, bin cards as well as dispensing registers to reconcile stock and funds. When explaining how medicine auditing is conducted, the district managers said:

“ … *… as CHMT we have a team for conducting medicine audit, the team visits the facilities to observe whether the medicine received were prescribed to the patients or there are some medicine which entered into unsafe pockets, we go through dispensing registers and compare with the existing stock level at the facility. At the end of the day, we make reconciliation and realize if they are smart or there is misappropriation* … .” IDI, CHMT, Dodoma.

Another form of medicine auditing reported by the district managers are unannounced (special) audits. Unannounced audits happen infrequently in the districts and were only mentioned in two out of the four districts visited. The district managers reported that they conduct special auditing to assess the existing medical stock or under suspicion of medicine fraud in the healthcare facilities. Unannounced auditing was done to identify shortcomings facing the facilities so that district managers may make a decision, offer advice, or take appropriate action.

### Performance accountability

Among the key performance indicators of Jazia PVS are increased availability of essential medicines at the facility and product delivery time at the district headquarter. The contracted vendor has to deliver the consignment at the district headquarters, while staff at the facilities are responsible for the pick-up of the consignment. Jazia prime vendor technical committee and regional programme managers monitor closely the performance of the vendor. In case of failure to meet the contractual agreements, regional authorities initiate a discussion with the vendor to remedy the situation. Continued disrespect of contractual obligations can lead to suspension or termination of the contract. During field work it was noted that participants’ experience on the vendor and healthcare facility performance was based on feedback information given to them during quarterly monitoring and evaluation activities coordinated by the regional prime vendor coordination office.

#### Medicine availability

The regional prime vendor coordination office reported that medicines availability and stock adequacy at health facilities were assessed quarterly each year as part of Jazia PVS performance assessment. Across facilities and districts in the two regions, the study participants felt that the prime vendor system has increased the availability of medicines at the facilities. The regional and district managers reported that the availability of tracer medicines for some facilities was 90 to a hundred percent. One of the district officials elaborated:

“ … .. *in the last financial year 2017/2018, I participated in the stocktaking exercise for the whole district, I visited all the healthcare facilities, dispensary, health centre and district hospital, I found availability of medicines was satisfactory, like ninety percent* … ..” IDI, District Manager, Morogoro.

Another district officer explicitly mentioned the upward trend in tracer medicines availability after the introduction of the Jazia prime vendor system;

“ … .. *after implementation of the system, availability of medicines increased up to seventy-five percent, then it reached eighty percent, from there up to eighty-five percent, then ninety, and right now the availability of essential medicines within the district is ninety-seven percent* … ..” IDI, CHMT, Dodoma.

Delivery time of consignment 

Regional and district managers reported that, the average delivery time was in most cases 14 days, with some hospital purchases delivered even within five working days. However, some delays were observed in moving the consignment from the district headquarter to the health facility level. Delays were linked to the lack of transport, communication challenges related to poor mobile phone signals, shortage of staff at the healthcare facility, and facility delays in processing orders to the districts.

### Procedure accountability

#### Adherence to standard operating procedures (SOPs)

Standard operating procedures were established to guide providers and district managers on procurement of commodities from the prime vendor (see supplementary material Box S1). In the SPOs it is stipulated that facility staffs are to consult the list of missing items ‘stock-out’ from MSD before placing orders to prime vendor. In addition to that they should ensure that they have sufficient funds in their bank accounts to pay for the medicines and that HFGC approves the use of available funds to procure required medicines. Moreover, SOPs indicate the obligation of the HFGC to inspect delivered commodities from the vendor, while the CHMT is responsible for inspection of the consignment at the district level. Lastly according to the SOPs, all communication with the vendor should be channelled through the district pharmacists. It emerged from the interviews that there were six different SOPs. In all healthcare facilities surveyed, it was reported that they do comply with the SOPs. This process is illustrated by the facility in-charge who said:

“ … .*we have a list of medicine stock-outs from MSD, we take the copy and request from the vendor. So we look at our facility bank account, how much is available, we call for HFGC meeting. We sit with them and prepare meeting minutes. Later on, I prepare a proposal for purchasing medicines from the vendor, and submit to the district pharmacist ..*..” IDI, Health Facility In-charge, Morogoro

In a discussion with the district pharmacists, they reported that they are responsible for consolidating and forwarding orders to the prime vendor. One of the district officials explained:

“ … *we consolidate facility requests, we enter all requests from our facilities in an excel sheet and forward it to the vendor. It may happen that eight facilities have brought their order out of quarterly schedule, we just request for them … …* .” IDI, CHMT, Dodoma.

#### Payment terms of the vendor

Contractual agreement specifies that the vendor should be paid within 22 days by the respective healthcare facility*.* District and regional managers reported that initially there were some delays in paying the vendor. In some cases, it took more than 30 working days to pay the vendor after delivering the consignment at the district headquarter. Initiatives were in place to address the problem of delayed payments including regular reminders to subordinates and order placement by facilities after reviewing available funds in their bank accounts. The main reasons for delayed payments mentioned were weak financial management skills and knowledge on rules and regulations pertaining to bank transfers, lack of cash flow, system closure to allow financial audits at the end of fiscal year, and the unfamiliar bureaucracy regarding transactions with the private sector. In fact, although reported vendor payment delays were rather short, they were particularly critical at the end of the fiscal year (30th of June in Tanzania) when facility bank accounts were usually frozen to allow a financial audit. One of the district officials when elaborating on the number of days it takes to pay the vendor said:

“ … *… there are payments which got stuck due to the government financial system. One consignment from 30the June has not yet been paid after entering a new financial year. The financial systems were still closed, till now they have not been opened [13*^*th*^*August 2018 date of interview], so we have delayed paying the vendor, we will finalize the payment once the systems are in operation … ”* IDI, CHMT, Morogoro

The representatives from the prime vendor confirmed that payment delays were sometimes a critical issue, at times payment delays could persist beyond 3 months.

“ … *… at the time we were in a meeting, it was observed that there are some districts which had gone up to a hundred and eighty days without making payments, that is one of the challenges we faced, of course in some places payments were not made according to the contract … ”* IDI, vendor representative, Dar es Salaam.

#### Peer cascade coaching

In each district, well performing healthcare workers are identified during supportive supervision and are offered responsibility for peer coaching (mutual learning partnership among healthcare facility staffs to help them unfold best practices and capabilities/creativity to address health facility challenges in order to improve service delivery). These coaches are selected based on their experience as well as knowledge of commodities management. They are further trained on integrated logistic supply at the district level and provided with tools to assist them when performing coaching in the assigned facilities. Coaches are assigned three to four dispensaries for peer coaching, ensuring that respective facility orders are well prepared and filled correctly in the so-called ‘request and reporting forms”. When discussing the role of the cascade coaching in the district they mentioned for instance:

“..*.after the establishment of prime vendor system, I think a lot of energy was directed to the coaches whom I have mentioned, ensuring that they identify/raise any challenges at the facility related to the prime vendor, for those which require district pharmacist to respond, then they communicate with the pharmacist ...*” IDI25, CHMT, Morogoro.

Theoretically, peer coaches are required to visit the dispensaries quarterly. However, during discussions with district managers, lack of transportation, lack of regional ownership as well as recognition of its importance and limited financial resources were reported as the main challenges for the efficient operation of cascade coaching. A few health facilities reported having not received any cascade coaching during the last financial year.

“ … .*.regarding cascade coaching, I think ownership by the district has been minimal, you may find that the district manager considers it to be the idea of HPSS. The support was given for the first two years, the following years it was left to the district to supervise themselves as part of their work … …* .” GD1, Regional implementers, Dodoma

#### Redistribution of Medicines

Reallocation of commodities to meet health facility needs was mentioned several times. Medicine reallocations at the facility are done whenever there is excess stock of certain medicines, close expiry dates and when there is an emergency need in any facility. For accountability purposes, each healthcare facility has a local issue voucher, which is used whenever commodities are transferred from one facility to the other. Facilities receiving the medicines do not make cash payment for the consignment received from another facility, rather, they return the medicines after purchasing. Supportive supervision are essential for medicines redistribution. Discussions with the district managers revealed that during supportive supervision, district managers take note of the facility with excess supplies or medicines close to the expiry date. In discussing this issue, district managers said:

“ … *as I said in the beginning, during supportive supervision you may find one facility has excess and the other one has none, we re-distribute, for example from facility A to facility B, from facility B to facility C* … … .” IDI7, CHMT, Dodoma.

## Discussion

The study aimed to analyse the role of accountability in the implementation of Jazia PVS in Tanzania. The findings show that several accountability mechanisms were of relevance for the successful implementation and operations of Jazia PVS. Financial accountability was positively influenced by the establishment of health facility bank accounts that were part of the DHFF mechanism. Successful operations of the Jazia PVS were possible as facilities had funds in their bank accounts and used the funds to purchase medical commodities from the prime vendor. Financial and inventory audits conducted at the facilities also contributed to the performance of the Jazia PVS. The team conducting the audits reviewed all the documents used to purchase complementary medicines, ensured that facilities use the prime vendor and instructed those which had not ordered from the dedicated vendor to purchase from the prime vendor. Good performance of the Jazia PVS was also attributed to adherence of standard operating procedures by health facilities and the vendor, assuring continued contractual obligations. Cascade coaching on the other hand contributed to the well-functioning of the Jazia PVS as healthcare staff had the opportunity to learn from colleagues on issues related to the complementary pharmaceutical supply system. Through such peer learning staff confidence increased, knowledge improved leading to better understanding of and compliance with Jazia PVS operation.

Regional, district, and healthcare facility staffs complied with the standard inventory and financial auditing procedures. It was therefore possible to track resource allocations, disbursements, and decisions on the utilization of financial resources during the implementation of Jazia-PVS. Findings of this study on inventory and medicine audits are consistent with those of other studies of the pharmaceutical supply system, which underlined the importance of financial accountability in ensuring high performance of the system [2, 4, 39]. For example, a study undertaken in Kenya revealed that accountability and transparency were key to the successful implementation of revolving fund pharmacies [4]. RFPs financial and medicines’ documents were audited on a weekly basis within the first 2 months of implementation and later on, audits were done on a monthly or bi-monthly basis [4]. Quarterly audits allow for transparency and close monitoring of the cash collected at the facility and procurement of medical supplies leading to improved availability of medicines. Auditing requires the use of standardized tools and systems for tracking products and financial information [30, 31, 39]. Tools should be designed in such a way that they capture relevant information in the supply chain ensuring medicines reach targeted clients without any leakage or misappropriation [30, 40, 41]. In the department of Veterans Affairs (VA) programme audits were conducted to examine whether prices paid for the purchases from the prime vendor were similar to those presented within the contractual agreement, and changes in the contract terms were effectively communicated to the right authorities [42]. In most cases whenever there were any inefficiencies or misconducts identified during auditing, measures were taken to improve the system [42, 43]. Regular audits and feedback to those being audited result in positive outcomes [43–45]. In Tanzania, audits conducted in the prime vendor pilot region resulted in significantly increased facility reporting rates to the district managers, whereas funds collected on user fees increased, and facility in-charges had to repay funds which were identified to be miss-used [43].

The findings on performance accountability show that prime vendor was typically able to meet performance targets, including delivery of consignment to the health facility within 14 days. However, there were some drawbacks in terms of the timely payment to the vendor. Similar to a study undertaken in a hospital setting in Tanzania to assess the performance of the private suppliers, the private vendors were able to meet contractual agreements, the order fulfilment rate was 90 % and the mean lead time of private suppliers’ to deliver pharmaceuticals to the health facilities was 10 days [46]. In the United States, prime vendor implementation lead to faster turnaround, higher order fulfilment rates, costs reduction, and increased satisfaction among program users [17]. Comparing the prime vendor system applied in Tanzania and the prime vendor implemented by the VA in the USA, Jazia PVS is largely paper-based, while the other entails considerable computerised record-keeping. It is envisioned that overtime Jazia PVS will be linked with the national electronic integrated logistics system, thus improving efficiency reducing the paper-based work. In addition, comparing the maturity of the US health system and supply chain, the VA prime vendor benefits from strong governance and accountability mechanisms. Procurement and contracting agreements between the government and the prime vendors have also been found to decrease stock-outs of medicines in Zambia [18]. Close performance monitoring and evaluation of the prime vendor system itself is equally important for the observation of contractual obligations by both contracted vendor and contracting regional authority. Non-adherence to contractual obligations by the private vendor may lead to contract termination. Non-adherence to contractual obligations by facilities, councils and the region may lead to loss of reputation and to loss of complementary supplies. In most cases, the Jazia PVS vendor and the respective regional authority adhered to the contractual agreement. The noteworthy exceptions were sporadic delays of consignments to councils, delivery from district level to health facility and payment of the vendor, implying both contract partners.

We have identified a number of procedure accountability activities undertaken in conjunction with the implementation of the Jazia PVS, including supportive supervision done by district managers and cascade coaching done by peer health facility personnel. Trap et al argues that adequate oversight and support to the healthcare facilities in store management, inventory controls, and record keeping is essential for the effectiveness of any supply chain [47]. Evidence suggests that close oversight of the medical commodities at the healthcare facilities helps in curtailing wastage of medicines which are about to expire, misappropriations or any incidence of pilferage; in turn, these measures improve efficiency and effectiveness of the pharmaceutical management as well as service delivery [39, 41, 47]. Close monitoring of activities during supervision increases accountability and problem-solving skills especially into primary healthcare facilities [45, 48, 49]. The content of supervision and provision of constructive feedback to those being supervised is crucial in strengthening the system as it improves professional practices [44, 48, 50].

Content and frequency of peer cascade coaching also contributed to the successful implementation of Jazia PVS. Ajeani et al. argue that cascade coaching done by frontline health workers has not only been found to improve medicine availability at the facilities but also teamwork and innovative local problem-solving approaches [51]. In Swaziland, close mentorship provided to the frontline staff at primary health facilities contributed to the improvement in stock management as well as reporting [52]. Both supervision and peer coaching face some challenges including lack of managerial training, shortage of time, official work responsibilities, and costs associated with travels [48, 53, 54].

Compared to previous vendor programs undertaken in Tanzania, accountability activities of Jazia PVS are anchored within the structures of the regional and district health administration for sustainability purposes and no new or parallel structures were created for implementation [23]. This decision was made to promote the sustainability of Jazia PVS. Implementation of Jazia PVS is in line with the Tanzania Health Sector Strategic Plans (HSSP) three (July 2009 – June 2015) and four (July 2015 – June 2020), which aim at ensuring a hundred percent availability of essential medicines in all primary health facilities in the country [55]. HSSP advocates for close collaboration with the private sector for effective delivery of healthcare services. HSSP also clearly stipulates that access to medicines could be achieved through new innovative approaches such as new contracting arrangements with the private sector [55].

The results of this study should be interpreted in the context of its limitations. The qualitative approach used cannot establish causality between implementation success and procedure and performance accountability. It rather offers a plausible overview on how accountability measures contributed to good performance. The number of analysts looking at the qualitative data analysis was limited, which possibly increased the potential for misinterpretation. A higher number of scientists analysing the phenomenon under investigation with multiple viewpoints has been found to be effective in producing more accurate results that are more accurate as well as lessening the chance of individual bias. However, it was not possible to include more analysts in the study. Moreover, the present results and analysis cannot be generalized to parallel broader health reforms happening in Tanzania, such as direct health facility financing, scale-up of improved community health funds, as well as improved facility financial accounting and reporting system. Boven’s accountability framework selected has not broadly been applied in the pharmaceutical supply chain and was not specifically tailored to this management area. Lastly we cannot weigh the contribution of the different elements examined in this study. For example, it is not possible to assess whether auditing is more important than shifting the decision power to healthcare facility level, which allows for financial autonomy and flexibility in the use of financial resources. Future studies could be designed to compare health facility data with the existing set of indicators used within the district health information systems (DHIS). Cascade coaching seems to improve knowledge and skills on medicine management, but more research on this approach is needed, especially on the selection of the coaches and content of the coaching materials as well as on its effect on accountability.

## Conclusion

This study shows that accountability mechanisms contributed to successful implementation of Jazia PVS in Tanzania. Specifically, auditing, well-structured financial reporting mechanisms on revenue and expenditure, standard operating procedures and cascade coaching accounted for the success of the system. However, sustained improvement in the availability of medicines at the primary healthcare facilities will also depend on correct quantification, timely delivery of consignments to the facility level and prompt payment to the contracted vendor. HFGCs have crucial role in decision making regarding health facility commodity needs and fund use. Therefore, capacity building for their members is important in strengthening financial management capacity. In this regard, one could argue that strong accountability and transparency in any intervention targeting the pharmaceutical supply chain is crucial. In conclusion, study provides some evidence that financial, performance, and procedure accountability measures play an important role for the successful performance of Jazia PVS in Tanzania, a complementary supply chain intervention.

## Supplementary information

**Additional file 1**: **Table S1.** Categories of respondents at the regional, district and facility level. **Table S2.** Respondents at the national, regional, and district level. **Box S1.** Jazia Prime Vendor System (Jazia PVS).

## Data Availability

The dataset supporting the results, discussion and conclusions of this paper is owned by Ifakara Health Institute (IHI) together with Health systems governance for an inclusive and sustainable social health protection in Ghana and Tanzania funded by the Swiss Programme for Research on Global Issues for Development (r4d programme, phase one) and available upon request.

## Abbreviations

ADDOs: Accreditation of drug dispensing outlets
CHF: Community Health Fund
CHMT: Council Health Management Team
CHSB: Council health service board
CMSs: Central Medical Stores
DHFF: Direct health facility financing
DHIS: The district health information systems
DMO: District medical officer
ELCT: Evangelical Lutheran Church Tanzania
FGDs: Focus group discussions
GDs: Group discussions
HFGC: Healthcare facility governing committee
HPSS: Health promotion and system strengthening
HSSP: Tanzania health sector strategic plans
IDIs: In-depth interviews
Jazia PVS: Jazia Prime Vendor System
LMICs: Low and middle-income countries
MEMS: Mission for Essential Medical Supplies
MSD: Medical store department
MUHAS: Muhimbili University of Health and Allied Sciences
NHIF: National Health Insurance Fund
PEPFAR: President’s Emergency Plan for acquired immune deficiency syndrome Relief
PO-RALG: President’s Office for Regional Administration and Local Government
PPP: Public-private partnership
RHMT: Regional health management team
SOPs: Standard operating procedures
SPHSS: School of Public Health and Social Sciences
Swiss TPH: Swiss Tropical and Public Health Institute
UHC: Universal Health Coverage
USA: United States of America
VA: Veterans Affairs

## Footnotes

1 In Tanzania, primary healthcare facilities have no financial autonomy; cost-sharing funds (user fees, basket funds, insurance reimbursements) are managed and controlled at the district level. In 2018, the government undertook some initiatives, allowing for fiscal decentralization. All public primary facilities were directed to open a bank account with a local/nearby bank and were approved by the Bank of Tanzania and cost sharing funds are deposited in the accounts (See Kapologwe et al. 2019).
