# Supplementary material for: The role of accountability in the performance of Jazia prime vendor system in Tanzania
Source: J Pharm Policy Pract. 2020 Jun 8;13:25. doi: 10.1186/s40545-020-00220-8 (PMC7278176; doi:10.1186/s40545-020-00220-8)
Supplement: Supplementary file 1 — Additional file 1: Table S1. Categories of respondents at the regional, district and facility level. Table S2. Respondents at the national, regional, and district level. Box S1. Jazia Prime Vendor System (Jazia PVS). [file 40545_2020_220_MOESM1_ESM.docx]

**Supplementary Material**

**Table S1: Categories of respondents at the regional, district and facility level**

| Region  Council  Category of Respondent | Dodoma Region | | | | | | Morogoro Region | | | | | |
| --- | --- | --- | --- | --- | --- | --- | --- | --- | --- | --- | --- | --- |
|  | Kondoa DC | | | Bahi DC | | | Ulanga DC | | | Kilosa DC | | |
|  | FGD | IDI | GD | FGD | IDI | GD | FGD | IDI | GD | FGD | IDI | GD |
| Council health management team (CHMT) |  | 2 |  |  | 1 | 1 |  | 1 |  |  | 2 |  |
| District accountants/ auditors |  | 1 |  |  |  |  |  | 1 |  |  | 1 |  |
| District procurement managers |  |  |  |  |  |  |  | 1 |  |  | 1 |  |
| Health service providers |  | 1 |  |  | 1 | 1 |  | 2 | 3 |  | 3 |  |
| Health facility governing committee (HFGC) | 5 |  |  | 3 |  |  | 2 |  |  | 4 |  |  |
| Council health service board (CHSB) |  | 1 |  |  |  |  |  |  |  |  |  |  |
| Total | 5 | 5 |  | 3 | 2 | 2 | 2 | 5 | 3 | 4 | 7 |  |

**Table S2: Respondents at the national, regional, and district level**

| Representative (s) | IDI | GD |
| --- | --- | --- |
| Health Promotion System Strengthening (HPSS) | 3 | 2 |
| President’s Office for Regional Administration and Local Government –PO-RALG (Jazia coordinating office) | 1 |  |
| President’s Office for Regional Administration and Local Government –PO-RALG (Procurement department) | 1 |  |
| Jazia PVS Regional Coordination office | 2 |  |
| RHMT members (Regional Pharmacists /Regional medical officer) | 2 |  |
| Prime vendor representative | 1 |  |
| Jazia PVS Consultant | 1 |  |
| Total | 11 | 2 |

**Box S1: Jazia Prime Vendor System (Jazia PVS)**

|  | **Description** |
| --- | --- |
| Jazia –PVS | - Jazia PVS is public-private partnership (PPP) initiative between the regional health authorities and private suppliers that aims at improving the availability of medicines, equipment, and supplies by consolidating and pooling orders for supplementary medicines from all public healthcare facilities at the district level and then purchasing them from one contracted single whole supplier ‘*the Prime Vendor*’ (22) for the duration of two calendar years (Figure, 1). Furthermore, along with the implementation of Jazia PVS, capacity-building, peer coaching, and auditing interventions were strengthened in order to improve accountability (23). Moreover, public financial management was reviewed and revised to standardize and simplify procedures and transactions. Additionally, the system is anchored in the structures of the regional health administration and is overseen, supported, and managed by mandated administrative structures |
| Mandated administrative structures | - Each region has a Prime Vendor coordinating office responsible for supportive supervision, training (on the rational use of medicines, ordering and reporting); monitoring of system performance and overseeing implementation of the system standard operating procedures (SOPs). The coordination office works closely with the Health Promotion and System Strengthening (HPSS), President Office Regional Administration and Local Government (PORALG), Regional Health Management Teams (RHMT), Council Health Management Teams (CHMT), prime vendor and healthcare providers. - Regional Prime Vendor Technical Committee (RPVTC). This is responsible with the provision of advice to all the technical and administrative issues including identification of the prime vendor, review of system performance and actors compliance with the contractual agreement. Meetings are held twice a year, though if the need for a meeting arises then the secretary may call for a meeting. The technical team is answerable to the tender board and to the regional administrative secretary (contract holder) - Regional Prime Vendor Tender Board (RPVTB) oversees the operations of the system, links to the technical committee, oversees transparency in the selection of the prime vendor. Regular meetings are held twice a year, but in case of anything, the board secretary may call for a meeting. After the rollout, the tender board has been anchored within the government structures to ensure compliance with the Public Procurement Act. |
| Other supporting mechanisms | - Ad hoc Regional Bids Evaluation Committee (EC). This is usually established whenever the need arises to evaluate the proposals of prequalification of suppliers and during the bidding evaluation process, in which prime vendors are selected. |
| Operating procedures (6) | - Jazia PVS has standard operating procedures guiding the process and the purchase of medicines from the prime vendor when these are either out of stock, in short supply, or not stocked by medical store department.  1. In each quarter health facility staff order commodities from CMS, upon delivery of the order, a list of missing items has to be presented to the facility. In turn, facilities review the list of missing items and prepare commodities (quantification) to be purchased from a prime vendor, conditioned on available funds at the facility bank account. 2. The order is shared with the district pharmacist, who consolidates the lists from other facilities and then forwards the consolidated order(s) to the vendor. 3. The prime vendor delivers the consignments at the district‘s headquarters, for inspection by district pharmacists and members of council health management team (CHMT). 4. Each facility is informed to come and collect its consignment at the district level. At the facility, consignment is then being inspected by the health facility governing committee (HFGC) 5. The facility in charge and the committee issues a cheque to pay the vendor, it is reviewed by district executive director (DED) or the district medical office (DMO), before being deposited into the vendor’s bank account. 6. All communications for the procurement, problems with the consignment and related issues are directed to the district pharmacists (DPharm). DPharm is responsible in all communication with the vendor and is answerable to the CHMT, technical committee and tender board. In the process of communication, DPharm has to inform the regional Jazia PVS coordination office. |
